# Trismus Nascentium

**Published:** 1884-06

**Authors:** A. W. Griggs

**Affiliations:** West Point, Ga.; Professor Emeritus of the Principles and Practice of Medicine in the Atlanta Medical College


### TRISMUS NASCENTIUM.

BY A. W GRIGGS, M D., OF WEST POINT, GA.

Professor Emeritus of the Principles and Practice of Medicine in the Atlanta Medical College.

The *American Journal of the Medical Sciences*, January, 1884, contains a very elaborate and highly instructive article on Trismus Nascentium, from the graceful pen of J. F. Hartigan, M.D., Washington, D. C., in which he ably discusses "its history, prevention, cause and cure," illustrating by cases, post-mortem examinations, etc. He supports the theory of the far famed gynæcologist, the good and great J. Marion Sims, M.D. (now deceased), who was fully of the opinion "that trismus nascentium is a disease of centric origin, depending upon a mechanical pressure exerted on the medulla oblongata, and its nerves; that this pressure is the result, most generally, of an inward displacement of the occipital bone often very perceptible, but sometimes so slight as to be detected with difficulty; that this displaced condition of the occiput is one of the fixed physiological laws of the parturient state; that when it persists for any length of time after birth, it becomes a pathological condition, capable of producing all the symptoms characterizing trismus nascentium, which are relieved simply by rectifying this abnormal displacement, and thereby removing the pressure from the base of the brain." Dr. Sims argued that the dorsal decubitus of the infant not only encourages but often causes the development of the symptoms, but further remarks, that by the prolonged lateral decubitus, the opposite relation may exist, the occiput becoming external to the parietal, and thus produce the pressure. Many years ago, I became greatly interested in a discussion on this subject between Dr. Sims, then of Montgomery, Ala, and Dr Jno. M. Watson, Professor of obstetrics and the diseases of women and children in the Medical Department of the University of Nashville. Dr. Watson advocated the prevalent idea, that trismus is caused by some morbid condition of the umbilicus. Dr. Sims's theory was novel, and its very simplicity commended it so strongly to my mind, that I was ready to embrace it, but determined to wait and watch every case coming under my care, and thus learn what I could at the bedside. It was not long before I became acquainted with the views of W. O. Baldwin, M.D., of Alabama, and a new direction was given to my thoughts and inquiries by this learned gentleman. Dr. B. maintains that trismus and tetanus are the same; holds to the umbilical theory, and teaches that the symptoms are to be explained upon the principle of "the reflex function of the excito-motary nerves." If trismus nascentium and tetanus are not identical, they present great similarity, and have many points in common. The modes of invasion and the subsequent phenomena are the same in the two; the modes of death are alike in both; each is more frequent and fatal among the negroes; and the same climatic or atmospheric influences predispose to both, under like circumstances. The only differences are declared by the post-mortem appearances, and I believe that this is easily accounted for if we will consider the physiological differences in the infantile and adult brain and nervous systems. In the adult the brain is the controlling power over the whole nervous apparatus; sways a pleasant mastery over the diversified functions during health, and readily recovers from very great shocks, and sometimes from severe structural lesions. There is much less controlling power in the brain in early childhood; there is a want of ability to adequately modify the morbid spinal reflexes when they exist, therefore children are more subject to convulsive attacks, and yield more readily to shock. Mental impressions often prove to be quite serious with them. I knew a little girl thrown into convulsions by a slight scratch from a cat, and another which was completely convulsed at the sight of a pair of dental forceps. Infants are more prone to cerebral disturbances and diseases, and these are more necessarily fatal than in after life. If we proceed upon the idea, that all the functions are performed through the agency of the nerves and their centers, and understand the great part played by reflex action, it will not be difficult to see, that *morbid impressions* can be made through this medium, upon organs not *primarily* involved. If this *morbid reflex* excitement be sufficiently intense and continued, the persisting perversion of functions may result in structural disease. The stimulus may exist in the disordered action of some organ, and reflected through a nerve center, produce an exaggerated or morbid sympathy; or again, the centripetal or centrifugal nerve filaments may be in a preternatural state of irritability; or the center of reflexion, from some intrinsic cause, be unable to transmit otherwise physiological impressions, and thus *morbid reflex* excitability may originate, or reflex paralysis occur. Tetanus is mostly due to *morbid reflex action*, produced or brought about by wounds, especially of the extremities. The phenomena arise from the effects of certain poisonous agents; such as strychnia, brucia, the St. Ignatius bean, etc. They sometimes arise from the cholera poison, and they sometimes are developed when we are absolutely unable to divine the cause.

Two fatal cases are recorded by Aitken, which were produced by the effects of noxious gases in the bottom of an old vat; other cases have arisen from the ligation of arteries, the application of the scarificator, and even from the extraction of a tooth. When of unknown cause we call it idiopathic, when due to a wound, traumatic; in other cases we say that the patient is poisoned, when circumstances favor the conclusion. In those obscure cases called idiopathic, where there is nothing to enlighten us as to the *causa causans*, I am of the opinion that we should look *within* as well as without, for a clue to the subtle agent so potent for evil. The cause, in instances of the kind, may yet be found in some retained excrementum, acting as a poison, as is the case in some diseases, for instance, uræmic poisoning, in morbus Brightii.

In the traumatic, the commonest variety, the wound often heals, antecedent to the outburst of the dreadful train of the characteristic symptoms. I do not think it impossible, that in such cases, some morbid action is going on in the tissue elements of the cicatrix and adjacent parts, on account of the imperfect nutrition in the new structure, and consequent formation of unhealthy products, which either act as an irritant to the peripheral nerves, or undergo a process of zymosis, as is thought to be the case in hydrophobia. I feel satisfied that Dr. Baldwin is correct in his views as to the identity of trismus nascentium and tetanus in the adult. The attention of the reader has already been called to the physiological differences between the infant and adult brain, and it does not appear unreasonable that effusions and extravasations should take place in connection with the former, even more readily than congestions in the meninges of the latter. Aitken says that "in a few cases (of tetanus), the membranes of the brain have been found congested; but not in a greater degree than might have been predicated from the violent and long continued muscular action incident to the disease." That "in a fewer number of instances small patches of cartilages or of bony matter have been found on the spinal arachnoid membrane." "But as these are often absent, they are not essential conditions of the disease." Erichsen says that, "the morbid appearances found after death throw little light on the nature of this affection; it is quite clear, however, from these, that the disease is not of an inflammatory character; indeed, the only morbid condition that is usually found, is a degree of congestion of the brain and spinal cord, with some bloody fluids in the ventricles and in the subarachnoid space."

All authors agree that these post-mortem appearances are not constant, and therefore are not essential conditions. They are, in our opinion, mere results, mostly due to protracted muscular contraction. Now if such results occasionally occur in the adult from tetanic spasms, ought we not to expect them oftener and more marked in the child with trismus; the inference is plain and very clear to me. The focus of primary irritation in trismus is, in my humble opinion, usually at the umbilicus. The funis not being supplied with nerves, can not be urged as an objection, as it is accommodated with two arteries and a vein, and tetanus has often been developed from the ligation of important vessels. Trismus comes up generally after the funis drops off, and some morbid condition is left, which may escape notice until it is too late. Again the umbilicus may not present any morbid appearance, and trismus may come up as tetanus sometimes does after the healing of a wound. An open, ulcerated or suppurating navel alone, is not capable of inducing an attack, but proves an important factor. There are other conditions and surroundings necessary; these have been mentioned by various writers as consisting of atmospheric vicissitudes, insufficient ventilation, bodily uncleanness, bad management generally, etc.

The great frequency of abnormal conditions of the umbilicus in these cases doubtless formed the basis of the pathology of early writers. I have seen many cases of inflamed, ulcerated and suppurating umbilici, that did well and never produced any impression on the nerves or their centers, that was perceptible. Again, I have often observed fatal cases after the healing of the navel. So also when the healing process had been interrupted, and there was ulceration or suppuration. These I have observed when there was no discoverable displacement of the cranial bones, and I have seen others in which the displacement was all that seemed to be wrong or unusual. My son, Dr. J. W. Griggs, of this city, a few days ago, reported to me a case of trismus in a negro child, which he ascribes to an operation for the relief of imperforate anus. You see that it is possible for trismus to originate in several ways, or from different predisposing and exciting causes. The debility and nervous excitability present in this class of patients renders them fit subjects to the disease, when other predisposing and exciting causes exist.

What has already been said of those mysterious cases of tetanus equally applies to trismus nascentium.

I am satisfied that cases occur from displacement of the occipital or parietal bone, and oftener by the former, but I do not think that they can be fairly chargeable simply to the pressure exerted upon the medulla oblongata and its nerves. It appears to me that the child would die of compression of the brain, and the concomitant symptoms be promptly and early exhibited in the case. Rupture of the basilar artery produces almost immediate death. Pressure, from whatever cause, at the base of the brain, is marked by the symptoms of apoplexy and paralysis, and without hesitation or delay. Prof. A. L. Ranney, M. D., of New York city, in an excellent article published in the *Medical Record*, April 12th, 1884, says that "when a lesion is situated at the base of the brain and is sufficiently large to involve the motor tract of both hemispheres, the body may be completely paralyzed below the head, various cranial nerves, chiefly the 3d, 5th 6th and 7th, are liable to then exhibit the effects of simultaneous pressure upon them ; hence the general paralysis of the body is apt to be associated with paralytic symptoms confined to the face. Bilateral spinal lesions, when situated high up in the cervical region, may also cause a form of complete paralysis of the body, the so-called cervical paraplegia."

It is possible that *irritation* of the cerebral meninges may cause trismus, in cases in which the pressure is not sufficient to produce compression of the brain; or that morbid reflex excitement due to the irritation of some of the nerves of the scalp from pressure by the *border* of the occipital or parietal bone, particularly when the one overrides the other, may be the cause. The majority of the cases that have come under my care during a practice of thirty years, I consider to have been due to some morbid condition of the umbilicus. It is also proper to state that the children of primiparæ, in proportion to number, have not been found to suffer any oftener than those of the multiparæ, though their heads are subjected to more violent and continued pressure and are much misshapen at birth. I propose to relate two cases, as there seems to be an individual interest in each.

On July 19th, 1873, after an easy and natural labor Mrs. ——, (white, age 32) was delivered of a living male child which was quite delicate. The mother and child did well until the 6th day, when my attention was directed to the fact that something unusual was the matter with the child. It had been kept mostly on its right side resting on a soft pillow, and had been well cared for in every particular. The babe could not nurse though apparently anxious to do so. It was evident that trismus was setting in, the jaw stiffened, the thumbs flexed on the palms. The head was thrown slightly back and there was preternatural heat of the head and abdomen. Close and repeated examinations failed to reveal any displacement of the occipital or parietal bones. Attention was now directed to the umbilicus, which appeared to be healed, but there was tenderness over and around it, and some induration of the adjacent parts, especially above. Iced cloths were applied to the scalp and warm flaxseed poultices placed over the abdomen and frequently changed. In ten or twelve hours a considerable quantity of pus exuded from the interior of the navel, which I supposed came from the cavity of the umbilical vein. The patient was greatly relieved, the symptoms disappeared, and he made a slow recovery with great difficulty, as he was naturally very frail.

In June, 1866, a young colored woman on my premises, a primipara, after ten hours of natural labor, was delivered of a very delicate living male child. On the 7th day notice was given that the child was sick and could not nurse. This was a thoroughly marked case of trismus. There was an inward displacement of the occiput, whether it was cause or effect, could not be determined. The child had rapidly emaciated since the attack twenty-four hours previous, and was considerably shrunken. The postural treatment was without avail. By the way, the nurse informed me that the child had not passed water since its birth, and upon examination, the umbilicus was found to be open and suppurating and the urine was flowing from it, the urachus not having closed. The patient died on the following day. In conclusion, allow me to commend the article of Dr. Hartigan to the careful consideration of your readers.

